# In Vitro Comparison of the Dimensional Accuracy of Implant Impressions Using Custom Acrylic Trays and Prefabricated Self-Perforating Trays: Protocol for a Comparative Evaluation Study

**DOI:** 10.2196/79452

**Published:** 2026-02-19

**Authors:** Arti Agrawal, Sharayu Nimonkar, Surekha Godbole, Rushabh Parakh, Vikram Belkhode, Namita Zilpilwar

**Affiliations:** 1 Department of Prosthodontics and Crown & Bridge, Sharad Pawar Dental College, Datta Meghe Institute of Higher Education and Research, 202, SPDC building, DMIHER, Sawangi (Meghe), Wardha, Maharashtra, 442001, India, 91 7057088288; 2 Bharati Vidyapeeth Deemed University, Sangli, Maharashtra, India

**Keywords:** implant impressions, custom-made trays, prefabricated trays, dimensional accuracy, prosthodontics, dentistry

## Abstract

**Background:**

The accurate transfer of implant positions from the patient’s mouth to the laboratory is crucial for the successful fabrication of prostheses in implant-based prosthodontics. Making an implant impression is a crucial part of this procedure as it replicates the intraoral implant position and transfers it to the cast to be used in the laboratory for fabricating a passive fit prosthesis. The purpose of this study is to compare and assess the accuracy of implant impressions made with prefabricated self-perforating trays and custom acrylic trays.

**Objective:**

The primary objective is to compare the dimensional accuracy of implant impressions made using custom acrylic trays versus prefabricated self-perforating trays in an in vitro setting. The secondary objective is to determine whether the impressions made with prefabricated self-perforating trays achieve clinically acceptable accuracy.

**Methods:**

A partially edentulous mandibular model with implants placed at the second premolar and first molar region will function as a master model. Impressions will be made using the custom acrylic tray and prefabricated self-perforating implant impression tray. The cast obtained will be scanned, and an STL file will be generated to evaluate the accuracy of implant positions using HyperMesh software. Statistical analysis will be done with IBM SPSS Statistics at a 95% CI and 80% power.

**Results:**

The study aims to evaluate whether there is a statistically significant difference in the accuracy of implant casts obtained using prefabricated self-perforating trays versus custom acrylic trays. As of July 2025, the models have been prepared for further analysis. It is projected that data collection will be completed in May 2026, with results to be published in early 2027.

**Conclusions:**

For the success of implant dentistry, the precise fit of the final prosthesis has become a necessity. A mismatched framework may overload the implant, endangering its durability. To achieve a precise fit of the implant framework, a precise definitive cast is necessary, which depends on several factors, one of them being the implant impression tray selection. Traditionally, custom acrylic trays have been used. However, they are impractical to use in routine clinical practice due to certain disadvantages. A newer range of trays for the open tray impression technique is being marketed, and one would be able to reduce the additional expense and laboratory time associated with using custom acrylic trays by using this newer variety of self-perforating implant impression tray.

## Introduction

### Background

The accurate transfer of implant positions from the oral environment to a working cast is paramount for the long-term success of implant-supported prostheses. Any discrepancies in this transfer can lead to misfits, resulting in mechanical complications such as screw loosening, screw fractures, and biological issues like peri-implantitis. Among the various factors influencing the accuracy of implant impressions, the choice of impression technique and tray type plays a significant role [1].

The open tray (direct) impression technique is the gold standard for implant impressions. This technique involves the use of impression copings that protrude through openings in the tray, allowing them to be unscrewed and removed along with the impression, thereby minimizing repositioning errors [1]. In contrast, the closed tray (indirect) technique requires the removal and repositioning of impression copings, which can introduce inaccuracies, particularly in complex implant scenarios [2].

Custom trays, typically fabricated from autopolymerizing acrylic resin, are designed to fit an individual patient’s anatomy, ensuring uniform thickness of the impression material and enhanced rigidity. These characteristics contribute to the high accuracy of impressions made using custom trays. However, the fabrication process is time-consuming and may not be feasible in all clinical situations [3].

Prefabricated self-perforating trays can be used as an alternative to these custom acrylic trays, as they do not require laboratory fabrication and are readily available. These trays consist of a thin film on the occlusal surface that is perforated by the transfer during tray positioning. This helps in achieving a clean and precise impression without excessive impression material. Although they offer practical advantages, concerns remain regarding their stability, their adaptation to the arch form, and the resulting accuracy of the impressions.

Previous in vitro studies have explored the accuracy of various impression techniques and tray types. Patil et al [4] evaluated the precision of different splinting materials in open tray impressions and found that all tested materials produced casts that had measurements very similar to the reference model, with prefabricated pattern resin bars showing the highest accuracy. However, there is a paucity of data specifically comparing the accuracy of impressions made using custom acrylic trays versus prefabricated self-perforating trays.

Therefore, we designed this in vitro study that aims to perform a comparative evaluation of the accuracy of implant impressions made using custom acrylic trays and prefabricated self-perforating trays. By controlling variables such as impression material, implant position, and environmental conditions, this study seeks to provide objective data to guide clinical decision-making regarding tray selection in implant prosthodontics.

### Aim

The primary aim is to evaluate the accuracy of implant impressions made with custom acrylic trays and prefabricated self-perforating trays.

The secondary aim is to assess if there is a clinically acceptable accuracy of impressions made by prefabricated self-perforating trays compared to the master model.

## Methods

### Study Design

The in vitro study will be conducted at the Department of Prosthodontics and Crown & Bridge of the Datta Meghe Institute of Medical Sciences.

### Sample Size Calculation

The sample size was calculated using OpenEpi software (version 3.0) for comparison of two independent means at a 95% confidence level and 80% power, based on previously published data. A minimum of 8 samples per group was determined, resulting in a total sample size of 16 (Group 1: mean 73.31, SD 26.01; variance: 676.52; Group 2: mean 154.41, SD 74.64; variance: 5571.13).

This was done with reference to the study conducted by Bohner et al [5].

The formula for the same is:


$$N=\frac{(\sigma_{1}^{2}+\sigma_{2}^{2}/\kappa )(Z_{1-\alpha /2}+Z_{1-\beta }{)}^{2}}{\Delta^{2}}$$

The notations for the formula are:

N=sample size

σ1=standard deviation of Group 1

σ2=standard deviation of Group 2

*Δ*=difference in group means

κ=ratio=1

Z1 – α /2 = two-sided Z value (eg, Z=1.96 for 95% CI).

Z1 – *β* = power

N=(26.01 × 26.01) + (74.64 × 74.64)(1.96+0.84)^2^ / (−81.1 × −81.1)=8

The total sample size is 16, with 8 in each group.

**Figure 1.**
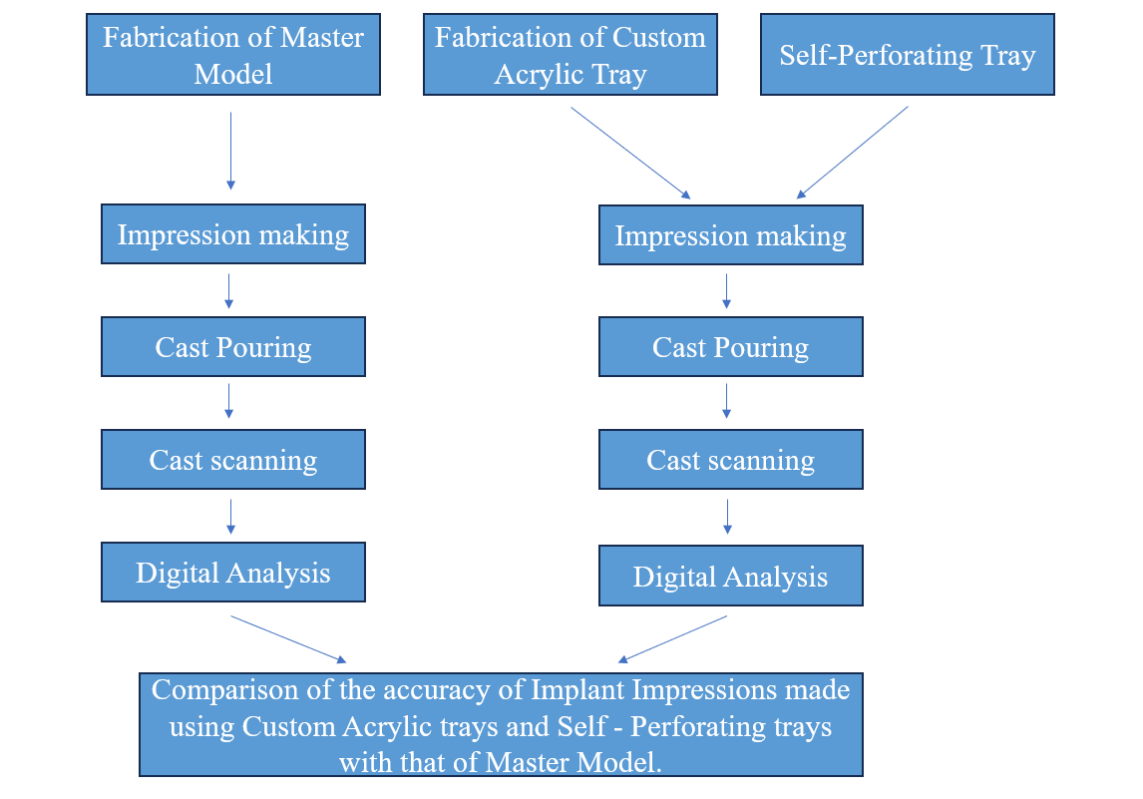
Schematic representation of the study procedure.

The study procedure is shown in Figure 1.

### Fabrication of the Master Model

**Figure 2.**
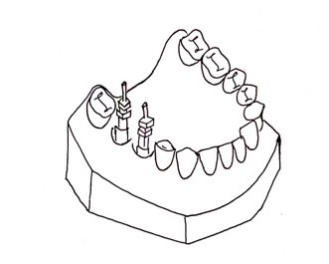
Fabrication of the master model.

A heat-cured acrylic resin model will be fabricated as the master model. This master model fabrication will be done by first creating a wax-up of the partially edentulous arch and positioning implant analogs using a dental surveyor. The assembly will be flasked, the wax will be boiled out, and heat-cured autopolymerizing polymethyl methacrylate (PMMA) resin will be packed into the mold. After curing in a water bath, the model will be deflasked, finished, and polished. This model will accurately replicate implant positions and serve as the reference for evaluating impression accuracy. Two implants will be placed parallel to each other in the second premolar and first molar region (Figure 2).

### Fabrication of Custom Acrylic Trays

To fabricate the custom acrylic trays from PMMA, which offers high rigidity and dimensional stability, a standardized procedure will be followed.

**Figure 3.**
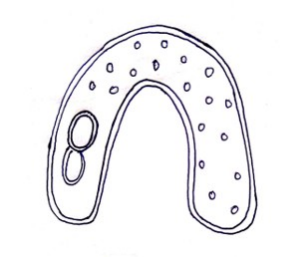
Fabrication of custom tray.

A denture flask will be used to create a mold. An impression will be made using irreversible hydrocolloid to obtain a space cast, and 4 mm of modeling wax will be applied over the master model to allow room for the impression material in order to build all of the custom trays with the same spacer thickness. To standardize the orientation of the custom trays on the model, the wax spacer will incorporate three tissue stops: two in the posterior region and one in the anterior region. A silicone putty mold will be made in the denture flask, and a tray of wax of consistent thickness will be applied over this space cast. The wax will then be removed by boiling it out for 7 to 10 minutes. Both halves of the flask will be covered with separating material. This flask mold will be used to create custom trays of the same thickness after PMMA resin has been mixed according to the manufacturer’s instructions. Once the polymerization is done, the trays will be taken out and will be bench cured for a full day before being used. A round bur will be used to create retention holes in each of the custom trays at evenly spaced intervals (Figure 3).

### Prefabricated Self-Perforating Trays

**Figure 4.**
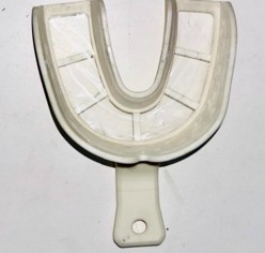
Prefabricated self-perforating tray.

Prefabricated self-perforating trays available under the brand name Cotisen Trays, which are made of rigid medical-grade polymer plastic, will be used in the test group. The occlusal surface of the prefabricated self-perforating tray is covered in patented plastic foil. After loading the tray with polyvinyl siloxane impression material, it will be positioned intraorally over the open tray impression heads and forced down crestally until the top of the impression pins can be seen through the clear foil. The impression pins will be pressed up against the tray until they pierce the foil and are visibly sticking out of it. After the impression material has set, an impression will be retrieved (Figure 4).

### Impression Making

The sample will be divided into two groups (A and B).

A total of 8 impressions will be made in each group—one using custom acrylic trays and the other using prefabricated self-perforating trays.

Polyvinyl siloxane medium-body impression material will be used to make impressions in both groups due to its excellent dimensional stability, high tear resistance, and superior elastic recovery—key factors for accurately capturing implant positions. Its minimal shrinkage and proven reliability make it the gold standard for implant-level impressions, especially with the open tray technique.

Impressions will be poured using type IV stone, and casts will be obtained.

### Scanning of the Obtained Casts

The casts will be scanned using a lab scanner (inEos X5), and an STL file representing the implant placement sites will be generated. This file will be imported into Altair HyperMesh 2021 (Altair Engineering Inc). HyperMesh will serve as the primary platform for refining the model, isolating the implants, and preparing the geometry for further analysis. The Circle Fit Tool in HyperMesh will be used to analyze the cross-sectional profiles of the implants, approximating their base geometry to ensure accurate center identification. Using the Node Tool, the geometric center node of each implant will be determined by fitting a perfect circle to the cross-section of the cylindrical body. This method will ensure precise identification of the implant’s central point.

### Measuring the Accuracy

**Figure 5.**
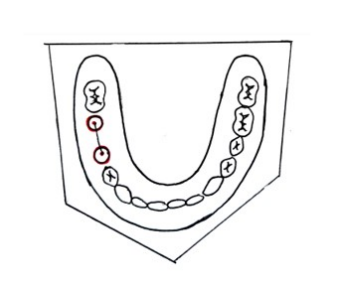
Measuring the distance between two implants.

After identifying the center nodes for both implants, the Measure Distance tool in HyperMesh will be used to calculate the distance between the two nodes. This measurement will represent the center-to-center distance between the implants. The process will be repeated multiple times to confirm consistency, and the results will be averaged to reduce the impact of potential variability or measurement noise (Figure 5).

### Evaluation of Accuracy

The accuracy of the implant impression tray will be evaluated for both groups. The experimental casts obtained from two distinct implant impression trays will be compared to the interimplant (analog) distance on the master model.

All impression-making, cast pouring, and scanning procedures will be performed by a single calibrated operator to ensure standardization and eliminate interoperator variability.

### Primary Outcome

The deviation in interimplant distance between experimental casts obtained from each tray type and the master model will be the primary outcome measure.

### Statistical Analysis

All analyses will be done using IBM SPSS Statistics for Windows (version 21.0; IBM Corp). The normality of data will be checked using the Shapiro-Wilk test as it is suitable for small sample sizes. In addition to that, Q-Q plots will be used for visual inspection of normality.

Intergroup comparisons will be performed using an independent samples *t* test if data are normally distributed; otherwise, the Mann-Whitney *U* test will be applied. Descriptive statistics will include mean and standard deviation or median and interquartile range depending on the underlying distribution of data.

### Ethical Considerations

The study has been approved by the institutional ethical committee of the Datta Meghe Institute Of Higher Education and Research (reference number DMIHER(DU)/IEC/2025/552).

## Results

The interimplant distance measured from casts obtained using custom acrylic trays and prefabricated self-perforating trays will be quantitatively obtained in this study and compared with the corresponding interimplant distance on the master model to determine the dimensional deviation for each tray type.

No funding was obtained for this study. Ethical approval was granted in February 2025. As of July 2025, the models have been prepared for further analysis. It is projected that data collection will be completed in May 2026, and results will be published early in 2027.

## Discussion

The accurate replication of intraoral implant positions is critical for the passive fit and long-term success of implant-supported prostheses [6]. The implant-bone connection necessitates a precise fit. A natural tooth’s periodontal ligament can move up to 100 μm, allowing for some misfitting to a fixed partial denture, although the maximum movement of an osseointegrated implant is 10 µm [7].

Misfit between the implant framework and underlying structures may result in internal stresses leading to both mechanical and biological complications such as screw loosening, screw fracture, or peri-implantitis [3,8]. Therefore, to create a successful implant-supported prosthesis, the first step is to accurately transfer 3D implant position and angulation from the mouth to the master cast using an impression [9].

One pivotal factor influencing this accuracy is the choice of impression tray—either custom-made or prefabricated self-perforating trays—used during the open tray impression technique.

Custom trays have long been considered the gold standard due to their ability to provide a uniform thickness of impression material and enhanced rigidity, reducing deformation during the impression procedure [10]. However, custom tray fabrication is time-consuming and may not be feasible in all clinical scenarios, especially where rapid impression-taking is necessary [11]. Prefabricated self-perforating trays offer a convenient alternative, allowing for immediate use without the need for laboratory fabrication. These trays are designed to facilitate direct access to the impression copings, essential for open-tray techniques. Despite their practicality, concerns remain about their stability, their adaptation to the arch form, and the resulting accuracy. This practical limitation has led to the need for a reliable alternative to custom-made implant impression trays [3].

Kwon et al [12] compared the dimensional accuracy of implant casts made with and without impression copings using an acrylic resin maxillary model with three implants. Two impression techniques were used to fabricate definitive casts. Centroid and long axis measurements of implant replicas were analyzed. Results showed significantly greater linear distortion in the no-coping group, especially at the first and second molar sites. The study concluded that impression copings improve cast accuracy, particularly when implants have greater interabutment distances.

Gupta et al [10] compared stock trays (plastic and metal) with custom trays and concluded that, when used with medium-viscosity impression materials, stiff nonperforated stock trays made of metal or plastic may serve as a substitute for custom trays for multi-implant impressions. Similarly, Pastoret et al [13] conducted a study to compare the dimensional accuracy of three impression techniques—a separating foil impression, a custom tray impression, and a stock tray impression. They concluded that the separation foil technique is a simple alternative to the custom tray technique for single tooth restorations, while limitations may exist for extended restorations with multiple abutment teeth [13]. Similarly, Goel et al [3] conducted an in vitro study comparing the accuracy of implant impressions made using custom trays (autopolymerizing and light-cured composite) versus specialized aluminum stock trays. Although statistically insignificant differences were observed among the groups, the light-cured custom trays demonstrated the highest accuracy, followed by autopolymerizing and stock trays. This reinforces the notion that custom trays can provide superior dimensional stability while also hinting at the potential clinical viability of stock trays under controlled conditions.

A review by Yasar et al [14] stressed that tray design must be evaluated alongside other variables such as implant angulation, number, and depth, which significantly impact impression accuracy.

Madhan et al [15] concluded that, despite advancements in materials and methodologies, some degree of dimensional distortion persisted, necessitating more studies to evaluate the transfer accuracy of implant impressions. The preliminary study by Spector et al [16] suggests that more research is required to identify methods that can reliably yield precise endosseous implant position recording.

In previous studies, when comparing casts made from stock versus custom trays to the master model, there was a slight statistical difference [17-19]. Although custom trays continue to demonstrate superior dimensional accuracy, prefabricated self-perforating trays offer a promising, efficient, and cost-effective alternative. Their clinical performance can be optimized through the use of appropriate impression materials and techniques. This study will further validate these findings and contribute to establishing evidence-based guidelines for tray selection in implant prosthodontics.
